# Integrated Degradome and Srna Sequencing Revealed miRNA-mRNA Regulatory Networks between the Phloem and Developing Xylem of Poplar

**DOI:** 10.3390/ijms23094537

**Published:** 2022-04-20

**Authors:** Changjun Ding, Tengfei Shen, Na Ran, Heng Zhang, Huixin Pan, Xiaohua Su, Meng Xu

**Affiliations:** 1 State Key Laboratory of Tree Genetics and Breeding, Key Laboratory of Tree Breeding and Cultivation of State Forestry Administration, Research Institute of Forestry, Chinese Academy of Forestry, Beijing 100091, China; changjund@126.com; 2 Co-Innovation Center for Sustainable Forestry in Southern China, Key Laboratory of Forest Genetics and Biotechnology of Ministry of Education, Nanjing Forestry University, Nanjing 210037, China; tengfeishen@njfu.edu.cn (T.S.); njlydxrn@163.com (N.R.); nanlinzhangheng@outlook.com (H.Z.); njfuhxpan@163.com (H.P.)

**Keywords:** secondary xylem, phloem, small RNAs, degradome, *Populus deltoides*

## Abstract

Lignin and cellulose are the most abundant natural organic polymers in nature. MiRNAs are a class of regulatory RNAs discovered in mammals, plants, viruses, and bacteria. Studies have shown that miRNAs play a role in lignin and cellulose biosynthesis by targeting key enzymes. However, the specific miRNAs functioning in the phloem and developing xylem of *Populus deltoides* are still unknown. In this study, a total of 134 miRNAs were identified via high-throughput small RNA sequencing, including 132 known and two novel miRNAs, six of which were only expressed in the phloem. A total of 58 differentially expressed miRNAs (DEmiRNAs) were identified between the developing xylem and the phloem. Among these miRNAs, 21 were significantly upregulated in the developing xylem in contrast to the phloem and 37 were significantly downregulated. A total of 2431 target genes of 134 miRNAs were obtained via high-throughput degradome sequencing. Most target genes of these miRNAs were transcription factors, including *AP2*, *ARF*, *bHLH*, *bZIP*, *GRAS*, *GRF*, *MYB*, *NAC*, *TCP*, and *WRKY* genes. Furthermore, 13 and nine miRNAs were involved in lignin and cellulose biosynthesis, respectively, and we validated the miRNAs via qRT-PCR. Our study explores these miRNAs and their regulatory networks in the phloem and developing xylem of *P.* *deltoides* and provides new insight into wood formation.

## 1. Introduction

The vascular system provides the transport system for water, minerals, and photosynthetic products in plants. It also supports the upright growth of plants and stores most of the biomass accumulated from photosynthesis. The vascular system consists of the xylem, which transports water, and the phloem, which transports nutrients [1]. Xylogenesis is considered as a sequential and complex process involving cambial cell mitosis, differentiation, secondary wall biosynthesis and lignification [2]. Cellulose is generated from glucose during the formation of β-1,4 glycosidic bonds to produce linear glucans, and is catalysed by cellulose synthase [3]. Cellulose is the most abundant natural organic polymer in nature [4]. Hemicelluloses are a type of heteropolysaccharide consisting of two or more monosaccharides with variable contents and structures [5]. As an important component of the secondary cell wall of plants, lignin is distributed in the cell walls of transport and lignified tissues and plays an important role not only in improving the water barrier function and mechanical strength of the cell wall, but also in increasing the resistance of plants to disease and stress [6,7].

Noncoding RNAs (ncRNAs) are a newly defined class of regulatory RNAs that do not encode proteins and were discovered in last century. They can interact with protein-coding genes to regulate gene expression. The ncRNAs can be classified into long noncoding RNAs (long ncRNAs) and small noncoding RNAs (sRNAs) according to their length [8]. The former mainly include circular RNAs (circRNAs) and lncRNAs, which are longer than 200 nt and usually display low sequence conservation and vary greatly between species; sRNAs include microRNAs (miRNAs) and small interfering RNAs (siRNAs), which are usually shorter in length but more conserved [8]. Plant sRNAs can be divided into two major categories, siRNAs and miRNAs, according to their biosynthetic processes and functional differences [9]. Regulatory sRNAs, represented by miRNAs and siRNAs, have been hot topics of research in the field of plant molecular genetics for almost two decades. SiRNAs generally originate from double-stranded RNA (dsRNA), are derive from variable locations and can be subdivided into heterochromatic siRNAs (hc-siRNAs), secondary siRNAs, and natural antisense transcript siRNAs (NAT-siRNAs) [9]. MiRNAs are highly evolutionarily conserved sRNAs of 20–24 nt in length and generated via single-stranded RNA folding to form hairpin RNA (hpRNA); miRNAs are derived from relatively fixed locations and degrade the transcripts of target genes or inhibit their translation by complementary base pairing with a target site, thereby regulating the growth and development of plants [9]. As miRNA research continues, increasing numbers of miRNAs and their target genes have been reported in plants; a large number of studies have elucidated the biosynthesis and functional mechanisms of miRNAs, and their biological functions have been widely revealed [10].

The biosynthesis of miRNA occurs via a multistep, multilevel reaction that includes the transcription of miRNA precursors, processing and transport of miRNAs, and assembly of the RNA-induced silencing complex (RISC); the first two steps occur mainly in the cell nucleus, while the RISC complex is formed in the cytoplasm [11]. The Transcription of MIR genes is mostly dependent on RNA polymerase II (Pol II) and is rarely mediated by RNA polymerase Ⅲ (Pol III) [12,13,14]. *MIR* genes are transcribed into primary miRNAs (pri-miRNAs) via Pol II- or Pol III-mediated catalysis. Pri-miRNAs have a stem-loop structure with a cap structure at the 5’ end and a polyA tail at the 3’ end and vary in length from a few hundred to thousands of nucleotides. Under the combined action of Dicer-like1 (DCL1), HYPONASTIC LEAVES1 (HYL1), and SERRATE (SE), pri-miRNA is further processed to form a precursor miRNA (pre-miRNA) [15]. Subsequently, the miRNA-processing machinery, the DCL1-HYL1-SE complex, then processes the pre-miRNA to form an miRNA/miRNA* duplex of approximately 21–25 nt in length [16]. The duplex is transferred from the cell nucleus to the cytoplasm via HASTY (HST) after being methylated by the transmethylation enzyme HUA ENHANCER 1 (HEN1) [17,18,19]. SE, HYL1, and HST have also been reported to have novel functions in miRNA biosynthesis. In addition to assisting DCL1 processing, SE can promote pri-miRNA degradation by recruiting Nuclear Exosome Targeting (NEXT) complexes and can affect the miRNA processing efficiency by promoting D-body formation [20,21,22]. In addition to forming homodimers to promote the efficiency and accuracy of DCL1 processing, HYL1 protects pri-miRNA from nucleic acid exonuclease attacks and maintains the dynamic balance of miRNA biosynthesis [23,24,25]. HST can also promote MIR gene transcription and processing by interacting with DCL1 [26]. Furthermore, newly identified processing factors such as the TREX complex (transcription/export), MOS4-associated complex 5 (MAC5), DAWDLE (DDL), PROTEIN PHOSPHATASE4 (PP4), and SMALL1 (SMA1), are involved in miRNA biosynthesis and processing, and most of these proteins regulate miRNA biosynthesis by interacting with DCL1 and HYL1 [25,27,28,29]. In the cytoplasm, Argonaute proteins (AGOs) bind to miRNA/miRNA* duplexes and release miRNA*, which is usually degraded [30,31]. AGO consists of the following four structural domains: the N-terminal, PAZ, MID, and PIWI domains. The PIWI domain displays RNaseH activity and is an essential functional component in the degradation of mRNAs by miRNAs [32]. When an miRNA binds to its target gene through complementary base pairing, the PIWI structural domain cuts the target gene at the site corresponding to positions 10–11 at the 5’ end of the miRNA, and the unprotected target gene is rapidly degraded by nucleases in the cell, thus losing its function [32].

Poplar is an important industrial timber species in the mid-latitudes. Species of section *Aigeiros* have become some of the most widely cultivated species worldwide due to their rapid growth and wide adaptability [33]. MiRNAs have been reported to function in the xylem of *P. tomentosa* and *P.* *trichocarpa*, but not in *P.* *deltoides* [34,35]. MiRNAs that can regulate wood formation have also been reported. In *P.* *trichocarpa*, miR397a was found to target a range of *laccase* (*LAC*) genes [36]. In *Arabidopsis thaliana*, miR397b negatively regulates *LAC4*. An overexpression of miR397b reduces lignin deposition and decreases fiber cell wall thickness [37]. In maize, miR528 can regulate lignin biosynthesis by negatively regulating *ZmLAC3* and *ZmLAC5* [38]. Wood formation is regulated by a three-level regulatory network of transcription factors with NAC-MYB at its core [39]. Numerous studies have reported that miRNA can target *MYB* and *NAC*. In *A. thaliana*, miR159 and miR399 was able to target *MYB*; in *Oryza sativa*, miR159 was found to target *MYB*; in *Gossypium hirsutum*, miR447, miR828, miR858, and miR 5255 was able to target *MYB*; in *A. thaliana* and *Z. mays*, miR164 was found to target *NAC*; in *Triticum aestivum*, miR164 was found to target *NAC1* [40,41,42,43,44]. However, it is unknown whether similar mechanisms exist in *P.* *deltoides* [39]. Here, we constructed six sRNA libraries for developing xylem and phloem derived from *P.* *deltoides* stems. We then systematically identified miRNAs involved in the developing xylem and phloem and further analysed miRNAs that may be involved in lignin and cellulose biosynthesis. This study will provide new insights into poplar wood formation.

## 2. Results

### 2.1. Construction of Small RNA Libraries and Sequencing

High quality RNA was extracted from developing xylem (X1, X2, and X3) and phloem (P1, P2, and P3) for sRNA sequencing. A total of 67,371,464 raw reads (3.37 G base) were obtained via small RNA sequencing, with mean Q20, Q30, and GC content values of 98.73%, 95.63%, and 50.65%, respectively. After filtering out low-quality reads (in which bases with a quality value of less than 20 account for more than 30% of the entire read), 275,357 reads in which N bases accounted for more than 10% of the read (unidentifiable bases accounted for more than 10% of the entire read), 106,181 reads with a 5′ adapter, 1,155,625 reads without a 3′ adapter, and 55,266 reads with poly A/T/C/G sequences, a total of 65,779,035 clean reads were obtained. Studies have shown that the length distribution of plant small RNAs ranges from 18 to 30 nt. A total of 52,233,007 reads of 18–30 nt in length were obtained using small RNA sequencing, including 23,684,340 and 28,548,667 reads specific to the developing xylem and phloem, respectively. Most of the sRNAs were 21 nt in length, and these sRNAs constituted 16.14% of the reads distributed within the six libraries (Figure S1). Finally, we used Bowtie to map the reads to the *P.* *trichocarpa* genome, and the X1, X2, X3, P1, P2, and P3 mapping rate values were 90.07%, 95.10%, 91.37%, 97.40%, 97.22%, and 97.39%, respectively. The above information indicates that the applied small RNA sequencing protocol met the requirements for the subsequent analysis. A brief summary and detailed information of the sRNA libraries are listed in Table 1 and Table S1.

### 2.2. Identification of MiRNAs Involved in the Developing Xylem and Phloem

**Figure 1:**
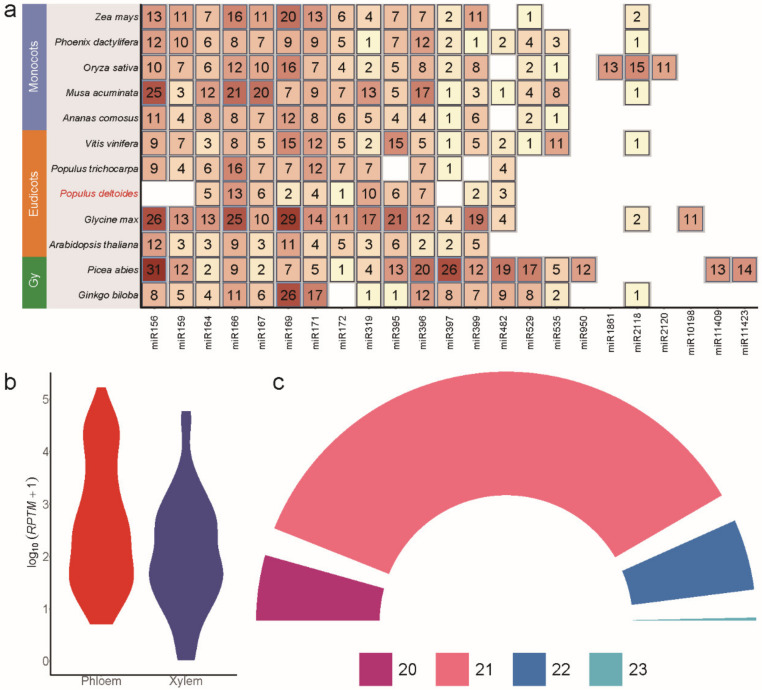
The miRNAs identified in the phloem and developing xylem of *P.* *deltoides* in this study. (**a**) Total numbers of known miRNA members in 12 plants. Only families with more than 10 members in at least one species are shown. Detailed information is provided in Table S2. (**b**) Expression value distribution of 134 miRNAs in the phloem and developing xylem of *P.* *deltoides*; the expression levels were log10 (*RPTM* + 1) normalised. The details of the expression of the 134 miRNAs are shown in Table S3. (**c**) Length distribution of the 134 miRNAs. The detailed sequences of the 134 miRNAs and their precursors are shown in Table S4.

A total of 194 miRNAs (97 mature miRNAs and 97 miRNAs*) were identified in *P. deltoides* according to the updated criteria for plant miRNAs, 96 of which were known miRNAs. We found that these known miRNAs belonged to 32 miRNA families. The largest miRNA family was the miR166 family, which had 13 members in the *P.* *deltoides* genome, followed by the miR319, miR396, miR160, miR167, miR395, and miR164 families, which had 10, 7, 6, 6, 6, and five members in the *P.* *deltoides* genome, respectively (Figure 1a, Table S2). After filtering out the miRNAs with low expression, we identified 134 miRNAs with high expression for the subsequent analysis, including 132 known and two novel miRNAs. Reads per ten million (*RPTM*) values were used to measure the expression levels of miRNAs, and the abundances of the miRNAs varied greatly. The *RPTM* values of pde-miR319d-mature, pde-miR166a-mature, pde-miR472a-mature, pde-miR396d-mature, and pde-482a-mature were greater than 10,000, and the values of pde-482a-star, pde-miR395f-star, pde-miR6427-star, pde-miR171b-star, and pde-miR476-star were lower than 100. The *RPTM* values also varied greatly between different members of the same miRNA family, as observed in the pma-miR159 family. The *RPTM* value of Pde-miR477c-mature in the phloem and developing xylem was greater than 500, but the *RPTM* value of Pde-miR477a-mature was lower than 300 (Table S3). In general, we found that miRNAs were highly expressed in the phloem relative to the developing xylem (Figure 1b). A total of 134 miRNAs ranging from 20–23 nt in length were identified; most of the miRNAs were 21 nt in length and accounted for more than 50% of the total miRNAs, which is consistent with the criteria established in previous studies (Figure 1c). Detailed information about the mature and precursor sequences of the 134 miRNAs is provided in Table S4.

### 2.3. Identification of Differentially Expressed miRNAs

**Figure 2:**
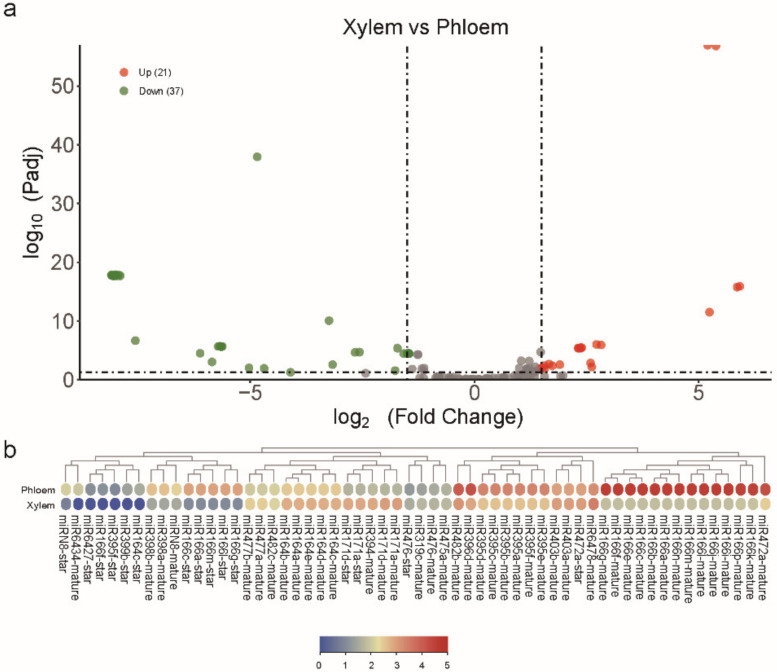
The DEmiRNAs found in the phloem and developing xylem of *P.* *deltoides* in this study. (**a**) Volcano plot of DEmiRNAs in the developing xylem relative to the phloem, with red and green dots representing up- and downregulated miRNAs, respectively. Detailed information on the log2 FC and Padj values of 134 miRNAs is shown in Table S5. (**b**) Clustering of the expression profiles of DEmiRNAs in the phloem and developing xylem; the expression levels were normalized by the log10 (*RPTM* + 1) normalised. The DEmiRNAs were divided into different clusters based on their expression profiles. Note: Due to some miRNAs in the same miRNA family having equal *y*-axis and *x*-axis, they overlap in Figure 2a. Based on the most recent plant miRNA annotation criteria, plant miRNA identification pipelines require the mapping of sRNA sequencing reads to the reference sequence and extending the reads that could be mapped to the reference sequence upstream and downstream by 150bp each, as putative miRNA precursors. In this process, there are a number of reads that may be mapped to multiple positions that share the same sequence, belong to the same miRNA family, perform similar functions, and are distinguished by appending a, b, and c to the nomenclature, respectively. Although they share the same sequence, in reality they are still different genes and, in theory, should be expressed differently, but at this stage, sRNA sequencing does not distinguish the extent to which each gene locus contributes to the overall expression, so the overall expression is used, leading to this phenomenon.

A total of 58 differentially expressed miRNAs (DEmiRNAs) were identified in the developing xylem in contrast to the phloem. Among the 58 DEmiRNAs, 21 miRNAs (21 known miRNAs) were more highly expressed in developing xylem than in phloem, while 37 miRNAs (35 known miRNAs and two novel miRNAs) were less highly expressed in developing xylem than in phloem (Figure 2a). DEmiRNAs accounted for 43.28% (58 out of 198) of all miRNAs. The top five upregulated DEmiRNAs in developing xylem relative to phloem were miR171a-mature, miR171d-mature, miR171a-star, miR171d-star, and miR394-mature. The log2 FC values of the five miRNAs were all greater than five. The most changed miRNAs were miR171a-mature and miR171d-mature, whose expression values in developing xylem and phloem were 1064.13 and 50.09, respectively (Figure 2b, Table S5). Their expression in developing xylem was twenty times higher than that in phloem, suggesting that these miRNAs may play an important role in developing xylem growth and development. The top downregulated DEmiRNAs in developing xylem relative to phloem were from the miR166 family and included miR166a-mature, miR166b-mature, miR166c-mature, miR166e-mature, miR166f-mature, miR166g-mature, miR166i-mature, miR166j-mature, miR166l-mature, miR166m-mature and miR166n-mature. The log2 FC values of the five miRNAs were all greater than seven (Figure 2b, Table S5). The miR166 family is highly conserved, and miR166 family members have been identified in several species by the efforts of researchers [45]. MiR166 has been found to be involved in seed, vascular, and root development. MiR166 family members showed dramatic changes in developing xylem and phloem expression, suggesting that they also play an important role in the growth and development of poplar [45].

### 2.4. Identification of MiRNA Target Genes via Degradome Sequencing

**Figure 3:**
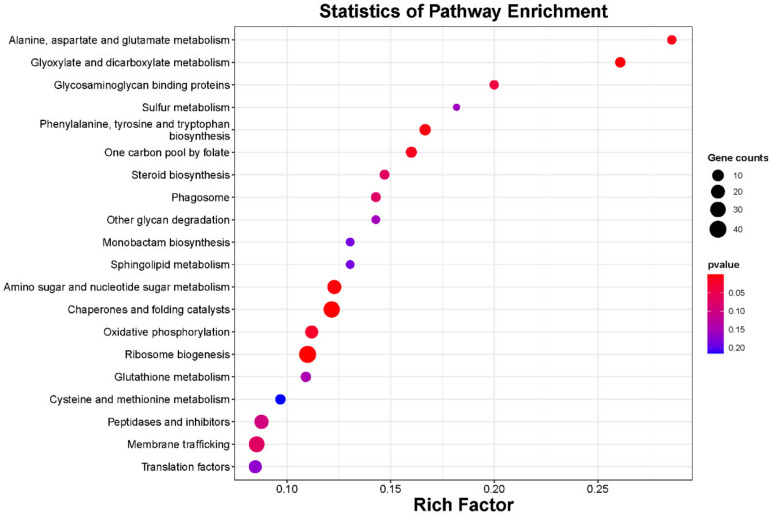
Statistics of KEGG pathway enrichment of miRNA target genes in the phloem and developing xylem of *P.* *deltoides*. The *y*-axis presents the KEGG pathway terms, and the *x*-axis presents the rich factors of the KEGG pathway terms. The calculation steps were as follows: the rich factor of certain KEGG pathways is equal to the number of genes in that pathway among miRNA target genes divided by the number of genes in the *P.* *deltoides* genome annotated in the pathway. The sizes and colours of the dots represent gene counts and *p* values, respectively. Larger dots indicate a greater number of genes, and deeper red coloration indicates a more significant *p* value.

A total of 2431 target genes of the 134 miRNAs were obtained via high-throughput sequencing. The miRNAs and their target genes could form 5180 regulatory pairs. The complete list of miRNA–mRNA regulatory pairs is shown in Table S6. Most miRNAs can target more than two target genes. For example, miR396 family members (miR396b-mature, miR396f-mature, miR396a-mature, miR396c-mature, miR396g-mature and miR396e-mature) could target approximately 30 different transcripts, while miR160 (miR160a-mature, miR160b-mature, miR160c-mature, miR160d-mature, miR160e-mature and miR160f-mature) and miR167 (miR167a-mature, miR167b-mature, miR167c-mature, miR167d-mature, miR167e-mature and miR167g-mature) family members could target approximately 20 different transcripts. Only 16 miRNAs appeared to target a single gene (Table S6). The target genes were enriched in nine KEGG pathways, and the top five terms were “chaperones and folding catalysts”, “ribosome biogenesis”, “glyoxylate and dicarboxylate metabolism”, “amino sugar and nucleotide sugar metabolism”, and “phenylalanine, tyrosine and tryptophan biosynthesis” (Figure 3). The top five terms among biological processes were “response to hormone”, “developmental process”, “DNA recombination”, “transcription, DNA-templated” and “histone modification”; the top two terms among cellular components were “COPI vesicle coat” and “spliceosomal complex”; and the top five terms among molecular function were “four-way junction helicase activity”, “lipid binding”, “unfolded protein binding”, “phosphatidylinositol phosphate kinase activity”, and “pyruvate kinase activity” (Figure S2). Table 2 lists several miRNAs target genes.

### 2.5. MicroRNAs Involved in the Regulation of Lignin and Cellulose Biosynthesis

**Figure 4:**
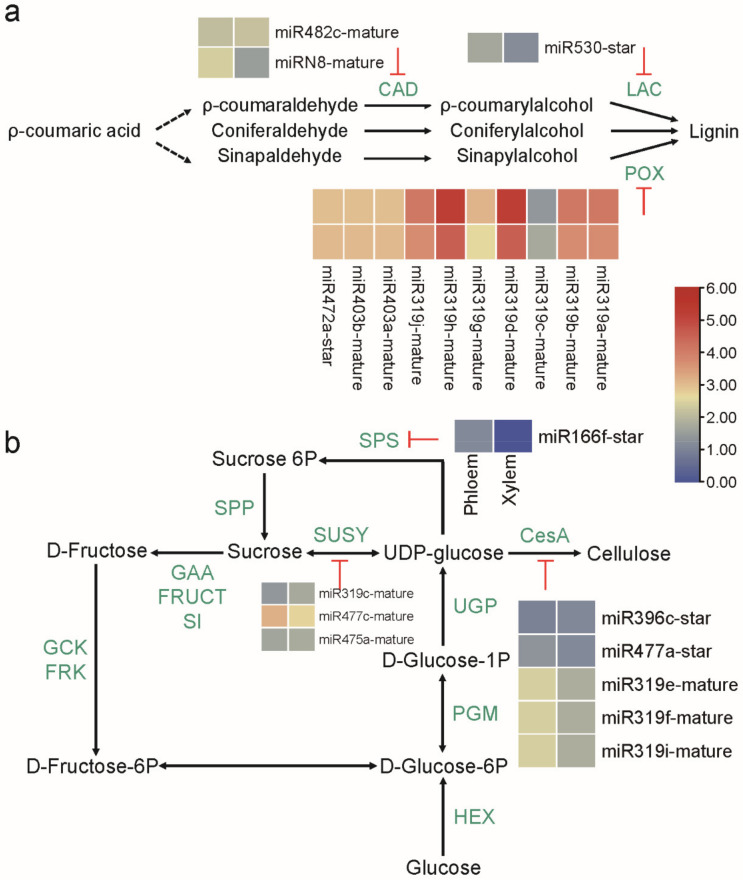
Typical pathways of lignin (**a**) and cellulose (**b**) biosynthesis in *P.* *deltoides*. Intermediates are shown in black, and the enzymes involved in each step are shown in green. The “T” arrows indicate the enzymes that could be cleaved by miRNAs supported by the degradome; expression levels was log10 (*RPTM* + 1) normalized.

A total of 13 miRNAs identified in this study may be involved in the lignin biosynthesis pathway, including 12 known miRNAs (miR482c-mature, miR530-star, miR319a/b/c/d/g/h/j-mature, miR403a/b-mature, and miR472a-star) and one novel miRNA (miRN8-mature). MiR482c-mature and miRN8-mature could target *CAD* genes, thereby inhibiting the conversion of ρ-coumaraldehyde, coniferaldetyde, and sinapaldehyde to ρ-coumarylacohol, coniferylalcohol, and sinapylalcohol, respectively. MiR530-star could target the *LAC* gene, and miR319a/b/c/d/g/h/j-mature, miR403a/b-mature, and miR472a-star could target *POX* genes, thereby inhibiting the polymerization of lignin monomers into polymeric lignin (Figure 4a). Among these 13 miRNAs, the top five highly expressed miRNAs were miR319d-mature, miR319h-mature, miR319a-mature, miR319b-mature, and miR319j-mature, all of which belong to the miR319 family. Additionally, miR319 family members could target *MYB* genes (Potri.001G036000.1, Potri.001G224500.2, Potri.003G189700.7) and the *TCP* gene (Potri.008G182300.4). MiR403 and miR472 family members could target *AGO* genes (Potri.012G118700.3, Potri.012G118700.4), which encode proteins that are essential for miRNAs function. MiR482 family members could target *R* genes (Potri.018G138500.2, Potri.003G201800.6), and an *LRR* gene (Potri.007G027500.4). MiR530 family members could target *bHLH* genes (Potri.014G099700.1, Potri.014G099700.2), a *GRAS* gene (Potri.003G110800.3), and a *WRKY* gene (Potri.018G107000.5). *Zinc finger protein* (Potri.005G115100.3) and *F-box* genes (Potri.018G048700.4) could be targeted by the novel miRN8 (Table S6).

A total of nine miRNAs may be involved in the cellulose biosynthesis pathway, all of which are known miRNAs. MiR319c-mature, miR477c-mature, and miR475a-mature could target *SUSY* genes, thereby inhibiting the mutual transformation of sucrose and UDP-glucose; UDP-glucose can also be transformed to cellulose when catalysed by CesA, and the *CesA* genes could be targeted by five miRNAs (miR396c-star, miR477a-star, and miR319e/f/i-mature). MiR166f-star could target *SPS* genes, thereby inhibiting UDP-glucose conversion to sucrose 6P (Figure 4b). Additionally, miR166 family members could target *WRKY* (Potri.010G160100.5), *P450 reductase* (Potri.005G153800.1), and *NAC* genes (Potri.002G037100.1). MiR475 family members could target *citrate synthase* genes (Potri.014G141900.1, and Potri.016G089300.1). MiR396 family members could target *GRF* genes (Potri.006G115200.2, Potri.014G007200.1, and Potri.007G007100.1), *SCARECROW-like* genes (Potri.016G009700.1, and Potri.016G009700.2), and an *AUX* gene (Potri.005G174000.1) (Table S6).

## 3. Discussion

### 3.1. Annotation of MiRNAs Involved in the Developing Xylem and Phloem of P. deltoides

**Figure 5:**
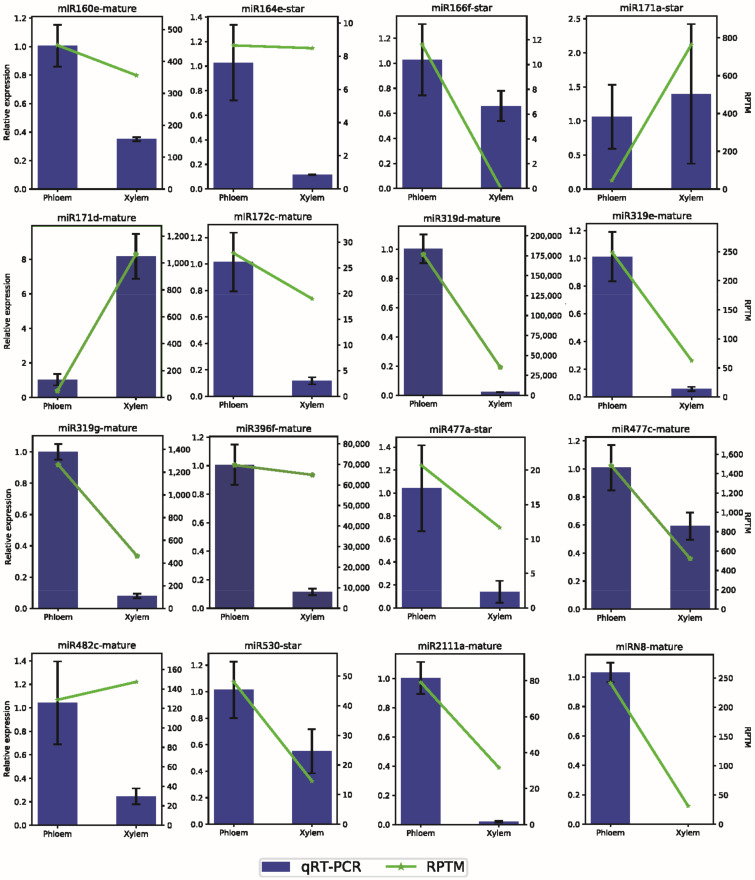
Tissue expression patterns of candidate miRNAs involved in lignin and cellulose biosynthesis validated by qRT-PCR.

A large number of studies have revealed the potential roles of miRNAs in plant growth and development and the responses to biotic and abiotic stresses, but no studies have yet revealed the functions of miRNAs in the developing xylem and phloem of *P.* *deltoides* [39]. In this study, we constructed six sRNA libraries (three for developing xylem and three for phloem) to systematically identify miRNAs involved in the developing xylem and phloem of *P.* *deltoides*. We obtained 67,371,464 raw reads via sRNA sequencing and 65,779,035 clean reads after quality control (Table S1). Studies have shown that the length distribution of plant sRNAs ranges from 18 to 30 nt. A total of 52,233,007 reads were obtained for miRNA prediction and analysis after the filtering of reads that were too long or too short (Table S1). Based on the most recent plant miRNA annotation criteria [46], a total of 134 miRNAs were identified as highly expressed in at least one library, including 132 known miRNAs and two novel miRNAs, among which six miRNAs (miR164c-star, miR166f-star, miR395f-star, miR399b-star, miR6427-star, and miR6434-mature) were only expressed in phloem, and their *RPTM* values were 32.58, 11.61, 7.79, 23.53, 10.79, and 93.30, respectively (Tables S2 and S3). We found substantial differences in the expression between developing xylem and phloem for 58 miRNAs, with 21 miRNAs significantly upregulated in developing xylem in contrast to the phloem and 37 miRNAs that were significantly down-regulated in developing xylem. We found that most miRNAs tend to be highly expressed in phloem (Figure 2b). We validated several miRNAs involved in lignin and cellulose biosynthesis via qRT-PCR, and the qRT-PCR and sRNA sequencing results of most of the selected miRNAs showed relatively high agreement (Figure 5). A total of 134 miRNAs were distributed among 32 different miRNA families. Most miRNA families presented more than one member in *P.* *deltoides* (miR160, miR166, miR167, miR319, and miR396), but some miRNA families were found to have only one member in *P.* *deltoides*, such as the miR172, miR394, miR475, miR476, miR530, miR6421, miR6427, miR6434, miR6459, miR6478, and miR7817 families (Table S2). The top five families were miR166, with 13 members, miR319 with 10 members, miR396 with seven members, miR160 with six members, and miR167 with six members. The length distribution of the 134 miRNAs ranged from 20 to 23 nt, and 21 nt miRNAs accounted for 79.10% of the total miRNAs, far exceeding the number of miRNAs of other lengths. A similar phenomenon is found in species such as *Pinus massoniana* (56.57%) and *Cinnamomum camphora* (more than 47%).

### 3.2. MiRNAs Families in Monocots, Eudicots, and Gymnosperms

MiRNAs belong to a class of non-coding genes and, similar to protein-coding genes, miRNAs are subject to constant evolution. Over the long period of plant evolution, many lineage-specific miRNA families have emerged, and these miRNA families have attracted much attention from researchers. For example, miR1515 is a species-specific miRNA present in *Citrus sinensis* [47]. Twelve plant species, including *P.* *deltoides*, were selected to investigate the presence of *P.* *deltoides*-specific, or genus *populus*-specific miRNAs, or eudicot-specific miRNA families. Among the 12 plants, the following five were monocots: *Zea mays*, *Phoenix dactylifera*, *Oryza sativa*, *Musa acuminata*, and *Ananas comosus*; the following five were eudicots: *Vitis vinifera*, *P.* *trichocarpa*, *P.* *deltoides*, *Glycine max*, and *A. thaliana*; and the last two, *Picea abies* and *Ginkgo biloba*, were gymnosperms.

**Figure 6:**
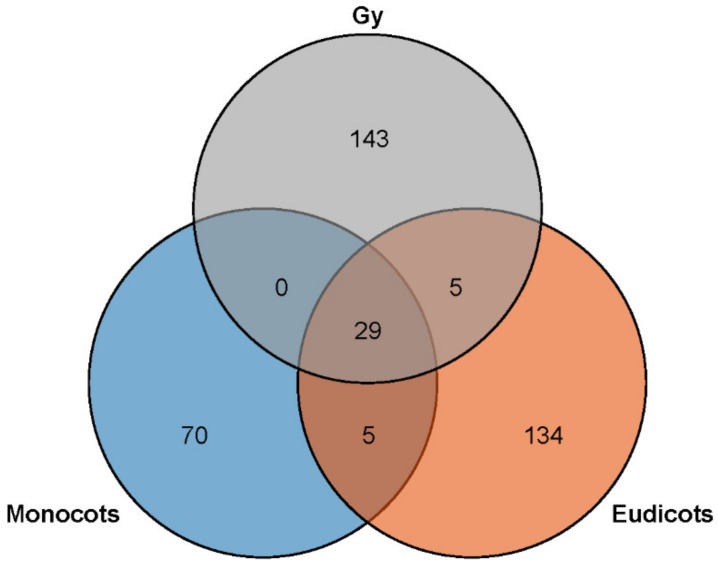
MiRNA families in monocots, eudicots, and gymnosperms.

A total of 386 miRNA families were found in 12 plant species. The eudicot *V. vinifera* had 152 miRNAs belonging to 43 different miRNA families; *P.* *trichocarpa* had 135 miRNAs belonging to 35 different miRNA families; *G. max* had 386 miRNAs belonging to 86 different miRNA families; *A. thaliana* had 152 miRNAs belonging to 82 different miRNA families; and *P.* *deltoides* exhibited 32 miRNA families, which were similar to those of *P.* *trichocarpa*, indirectly indicating that *P.* *deltoides* and *P.* *trichocarpa* are more closely related than the other plants (Table S2). The monocot *Z. mays* had 185 miRNAs belonging to 36 different miRNA families; *O. sativa* had 273 miRNAs belonging to 93 different miRNA families; *M. acuminata* had 210 miRNAs belonging to 31 different miRNA families; *P.* *dactylifera* had 128 miRNAs belonging to 28 different miRNA families, and *A. comosus* had 123 miRNAs belonging to 31 different miRNA families (Table S2). The gymnosperm *P.* *abies* had 547 miRNAs belonging to 171 different miRNA families, and *G. biloba* had 188 miRNAs belonging to 37 different miRNA families (Table S2). Eleven of the 386 families were present in all 12 plant species and are as follows: miR160, miR162, miR164, miR166, miR167, miR168, miR169, miR171, miR319, miR394, and miR396, all of which are highly conserved miRNA families (Tables S2 and S7). In addition, 18 miRNA families were present among gymnosperms, monocots, and eudicots. There were 70, 134, and 143 miRNA families that were specifically present in monocots, eudicots, and gymnosperms, respectively. Five miRNA families were present in both monocots and eudicots (miR530, miR5072, miR479, miR827, and miR158); five miRNA families were present in both gymnosperms and eudicots (miR4414, miR2111, miR2950, miR3627, and miR477); no miRNA families were exclusively present in gymnosperms and monocots; and 29 miRNA families were present across gymnosperms, monocots, and eudicots (Figure 6, Table S7). Most miRNA families (84.46%) were species specific, including 135 miRNA families present only in *P.* *abies*; 58 present only in *O. sativa*; 54 present only in *A. thaliana*; and 54 present only in *G. max* (Figure S3).

We found there were some differences in the miRNA family distribution and members among four species of *Populus* (*P.* *deltoides*, *P. trichocarpa*, *P.* *alba*, *and P. tremula*). *P.* *trichocarpa* had 135 miRNAs belonging to 35 different miRNA families; *P.* *alba* had 149 miRNAs belonging to 32 different miRNA families; *P.* *tremula* had 143 miRNAs belonging to 31 different miRNA families, and *P.* *deltoides* exhibited 32 miRNA families. There were 18 miRNA families present in all four species of *Populus* (*P.* *deltoides*, *P.* *trichocarpa*, *P.* *alba*, and *P.* *tremula*); one miRNA family (miR6459) present in *P.* *deltoides*, *P. trichocarpa*, and *P.* *alba*; three miRNA families (miR399, miR395, and miR6457) present in *P.* *deltoides*, *P.* *alba*, and *P.* *tremula*; six miRNA families (miR156, miR159, miR1448, miR397, miR390, and miR393) present in *P.* *trichocarpa*, *P.* *alba*, *and P. tremula*; three miRNA families (miR7841, miR828, and miR536) present in *P.* *alba* and *P.* *tremula*; one miRNA family (miR477) present in *P.* *deltoides* and *P.* *alba*; five miRNA families (miR476, miR475, miR530, miR6421, and miR2111) present in *P.* *deltoides* and *P.* *trichocarpa*; one miRNA family (miR1447) present in *P.* *trichocarpa* and *P.* *tremula*. Furthermore, four miRNA families (miR6434, miR6478, miR1446, and miR7817) were *P.* *deltoides*-specific; and four miRNA families (miR6456, miR1444, miR6425, and miR408) were *P.* *trichocarpa*-specific, (Figure S4). The top five miRNA families in *P.* *deltoides* were miR166, miR319, miR396, miR160, and miR164; in *P.* *trichocarpa* they were miR166, miR171, miR156, miR169, and miR164; in *P.* *alba* they were miR166, miR171, miR156, miR169, and miR167; and in *P.* *tremula* they were miR166, miR171, miR169, miR156, and miR167. Some miRNA families have similar members between the four species of *Populus*, such as miR166, miR160, miR168, miR390, miR394, miR396, and miR472, while some miRNA families are the opposite, such as miR1444, miR1446, and miR1447 (Figure S5, Table S8). The number of some miRNA families in *P.* *deltoides* was lower than in other species of *Populus*, such as miR156, miR390, and miR397. We think that this result is due to the fact that there were only two tissues in this study, whereas the miRNA data from other species of *Populus* originated from multiple tissues and differences in the depth of research on miRNAs in different plants.

### 3.3. Most Target Genes Are Transcription Factors

Two types of regulators play an important role in eukaryotic post-transcriptional gene expression, and are as follows: transcription factors and miRNAs, both of which perform regulatory functions at the transcriptional and posttranscriptional levels [48]. Transcription factors are a class of proteins with specific functions that initiate transcription by binding to the promoter region of a gene or recruiting other proteins to the promoter region [49]. 

**Figure 7:**
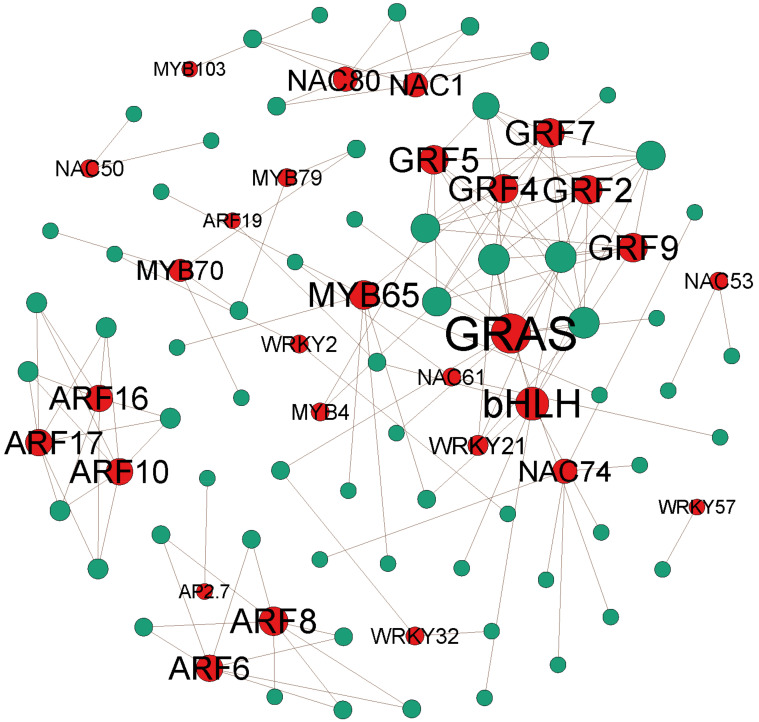
MiRNA-mRNA regulatory networks. The red and green dots indicate target genes and miRNAs, respectively.

MiRNAs are a class of small noncoding RNAs discovered in the last century that can directly degrade target mRNAs or inhibit the translation of target genes by binding to their 3’ UTRs [11]. Transcription factors and miRNAs have also been found to be mutually regulated, with transcription factors binding directly to the promoter regions of miRNAs and regulating miRNA biogenesis, while miRNAs can directly target the mRNAs of transcription factors, making it impossible for the proteins to perform their original roles [49]. In this study, the majority of identified miRNA target genes were found to encode transcription factors, including *AP2*, *ARF*, *bHLH*, *bZIP*, *GRAS*, *GRF*, *MYB*, *NAC*, *TCP*, and *WRKY* genes (Figure 7; Table S6). For example, miR172, targeting *AP2*, has been reported to be involved in floral development in *A. thaliana* and to be involved in nodule formation in *G. max* and *Phaseolus vulgaris* [50,51,52]. MiR160, targeting *ARF*, is reported to be involved in the vascular cambium initiation process in *P.* *tomentosa* and to be involved in root growth in *A. thaliana* [34,53]. In *A. thaliana*, miR159, targeting *MYB*, has been reported to be involved in seed germination [54]; in *G. hirsutum*, miR447 and miR5255, targeting *MYB*, have been reported to be involved in root and fibre development [43]. Furthermore, miR319, targeting *TCP*, is reported to be involved in regulating secondary growth in *P.* *tomentosa* and to be involved in floral and leaf development in *A. thaliana* [34,43].

### 3.4. Conserved MicroRNAs-mRNAs Modules Involved in the Biosynthesis of Lignin and Cellulose

A total of 13 miRNAs that may be involved in lignin biosynthesis in *P.* *deltoides* were identified in this study. MiR482c-mature and miRN8-mature, targeting *CAD* genes, inhibit the conversion of ρ-coumaraldehyde, coniferaldetyde, and sinapaldehyde to ρ-coumaraldehyde, coniferylalcohol, and sinapylalcohol, respectively. CAD is one of the key enzymes in lignin biosynthesis, producing precursors of lignin monomers (ρ-coumaryl alcohol, coniferyl alcohol, and sinapyl alcohol) [7]. A novel miRNA whose target gene was *CAD* was identified in *Artemisia annua* [55]; in addition, the level of Pde-miR5913 was shown to decrease after infestation with *Marssonina brunnea*, which led to an upregulation of its target gene *CAD*, assisting the plant in dealing with biotic stress [56]. MiR530-star, targeting *LAC*, inhibits the polymerization of lignin monomers into lignin. *LAC* has been reported to be regulated by miRNAs in *Pinus massoniana*, *Z. mays*, *P.* *trichocarpa*, *Linum usitatissimum*, and *Pyrus bretschneideri*, implying that the role of the miRNA-*LAC* module in lignin biosynthesis may be conserved in gymnosperms and angiosperms [36,38,39,57]. *POX* can be targeted by 10 miRNAs, most of which are members of the miR319 family. The target gene of miR3493b in *T. aestivum* encodes the peroxidase 52 protein, suggesting that this miRNA is also a conserved miRNA-mRNA regulatory module [58].

A total of nine miRNAs that may be involved in cellulose biosynthesis in *P.* *deltoides* were identified in this study. MiR319c-mature, miR477c-mature, and miR475a-mature can target *SUSY* by inhibiting sucrose conversion to UDP-glucose. SUSY is a key enzyme in plant sugar metabolism that catalyses the biosynthesis and breakdown of sucrose and plays an important regulatory role in starch and cellulose biosynthesis, fruit quality development, reproductive growth, and sugar signal transduction. In *Panicum virgatum*, miR156 has been reported to be a regulator of SUSY [59]. MiR396c-star, miR477a-star, miR319e-mature, miR319f-mature, and miR319i-mature can target *CesA* by inhibiting UDP-glucose conversion to cellulose. CesAs in plants are components of a cellulose synthase complex (CSC) that is localised on the cell membrane and contains six large subunits, each including at least three cellulose synthases [60]. The CSC contains a large number of proteins, including CesA proteins, cellulose-like synthase (CSL) proteins, and KORRIGAN proteins [61]. In *A. thaliana*, miR395a has been reported to regulate a transcript encoding cellulose synthase [62]. MiR166f-star can target *SPS* by inhibiting UDP-glucose conversion to sucrose 6P. SPS is widespread in plants and is a key rate-limiting enzyme in the biosynthesis of sucrose. The amino acid sequences of SPSs in plants are generally long, and these SPSs contain additional long amino acid sequences that can be subjected to posttranslational modifications that in turn regulate the enzyme activity of SPSs [63,64]. In *Sorghum bicolor*, a novel miRNA has been reported to target a transcript encoding sucrose phosphate synthase [65]. Furthermore, some miRNAs, such as miR166a-star and miR166e-mature, target *homeobox genes*, which have been linked to wood development. Overexpression of *ATHB8* (a *homeobox* gene family member) in *A. thaliana* accelerates lignin accumulation [66].

Conserved miRNAs-mRNAs modules involved in wood formation are also reported in other species of *Populus*. A study on the potential role of miRNAs in wood formation in *P.* *trichocarpa* showed that there were 57 miRNAs enriched in xylem, of which 11 miRNAs were enriched in mechanically treated xylem (MTX). MiRX50A, an MTX-specific miRNA, has been predicted to target *xyloglucan endotransglycosylase/hydrolase16* (*XTH16*) and is thought to play a critical role in G-fiber formation and function. The xylem-specific miRNAs, miRX50 and miRX87, may be involved in wood formation via targeting the NAC domain TFs NAC083 and NAC050, respectively. MiRX41 is an MTX-specific miRNA involved in wood formation via targeting *CSLD4*, which encodes cellulose synthase [35]. According to a study on the potential role of miRNAs in diverse phases of wood formation in *P.* *trichocarpa*, members of the miR858 family, miR858x and miR858y, directly regulate secondary wall biosynthesis by targeting *MYB35*, *MYB52*, *MYB63*, and *MYB83*. Novel-m0998-5p could target *MYB5*, which forms MYB–bHLH–WDR TF complexes to activate the *PAL* gene, which encodes the first enzyme of the lignin biosynthesis pathway. Novel-m0260-5p could target *C4H*; novel-m1190-3p could target *LAC2*; and miR397x could target *LAC11*. They are all related to lignin biosynthesis and deposition [67].

## 4. Materials and Methods

### 4.1. Plant Materials and Small RNA Sequencing

Phloem and developing xylem tissues were derived from three 10-year-old ramets of *P.* *deltoides* cv. ‘I-69′. The developing xylem was harvested separately from the vigorously growing stems of trees in mid-July of the current year. The bark was peeled to expose the youngest developing secondary xylem, which was gently scraped from the wood with a razor blade and used for RNA extraction. Three ramets of each clone represented three biological replicates. Total RNA was extracted from the developing xylem and phloem of *P.* *deltoides* using an RNAprep Pure kit (Tiangen, Beijing, China) according to the manufacturer’s instructions. In our previous publications, we described the methods applied for the construction and sequencing of small RNA libraries [39]. In brief, the concentration of total RNA extracted from the developing xylem and phloem of *P.* *deltoides* was measured using the Qubit^®^ RNA Assay Kit (Life Technologies, Carlsbad, CA, USA), and RNA integrity was assessed according to RNA Integrity Number (RIN). Six small RNA libraries were constructed using the NEBNext^®^ Multiplex Small RNA Library Prep Set for Illumina^®^ kit (NEB, Ipswich, MA, USA), and the six sRNA libraries were then sequenced using the Illumina HiSeq 2500 platform for 1 × 50 bp sequencing (Novogene, Beijing, China).

### 4.2. MiRNA Annotation Pipeline

The raw data of six small RNA libraries were used to assess sequencing quality with FastQC [68]. We used Cutadapt to filter out the reads that did not meet the criteria for plant small RNA [35], which were less than 18 nt or more than 30 nt in length, after using dnapi.py to obtain the adaptor sequences [69]. To increase the quality of clean data, we also filtered out reads containing more than one N (ambiguous base) base and required the quality values of all bases in the reads to be greater than 20. Studies have reported that small RNA fragments derived from rRNA or tRNA may interfere with the accuracy of miRNA annotation [70]. We used the Bowtie to remove reads that were derived from rRNA, tRNA, or organelle genomes, with one base mismatch permitted [71]. Then, miRNA annotation was performed using sRNAminer according to the most recent plant miRNA annotation criteria [46,72,73]. Briefly, miRNA/miRNA* duplexes do not contain a large loop, which limits the precursor and mature miRNA lengths to 300 nt and 20–24 nt, respectively; and miRNA/miRNA* duplexes contain up to five mismatches, only three of which are in the loop region. After removing miRNAs that were represented by less than 10 read counts in all samples, a total of 134 miRNAs were finally obtained. Known and novel miRNAs were identified based on comparisons with the plant miRNAs stored in the miRBase database, revealing 132 known miRNAs and two novel miRNAs. In this study, we used DEseq2 to perform the differential expression analyses of miRNAs. Firstly, we filtered out the miRNAs that were not expressed in all samples and used the *DESeqDataSetFromMatrix* function to construct a *DESeqDataSet* object; secondly, we used the *DESeq* function to perform differential expression analyses and used the *sizeFactors* function to calculate the scaling factor; thirdly, we used the *results* function to extract the results of differential expression analyses and used the *estimateSizeFactors* function to calculate the normalized counts of miRNAs. MiRNAs with a |log2 (fold change)| value of more than 1.5 and an Padj value of less than 0.05 were identified as differentially expressed [74]. The normalised expression levels of miRNAs were measured as *RPTM* (reads per ten million), which were calculated as follows:

(1) RPTM=miRNA read countstotal mapped reads*10^7

### 4.3. Identification of MiRNA Target Genes

The RNA isolated from the developing xylem and phloem was mixed together in equal amounts to construct the degradome library, which was subjected to 1 × 50 bp sequencing on the Illumina HiSeq 2500 platform (Lianchuan Bio, Hangzhou, China). The whole-genome sequence, coding sequence, and gene function annotations of *P.* *trichocarpa* v4.1 were retrieved from the Phytozome database [75]. The degradome sequencing data were then subjected to adaptor removal using fastp with the default parameters [76]. Finally, we used CleavelLand4 to address the sequencing data and obtain the target genes of miRNAs [77]. GO and KEGG enrichment analyses of miRNA target genes were performed via clusterProfiler [78].

### 4.4. qRT-PCR Validation

The reference genes selected for use in qRT-PCR validation in this study were *18S* and *EF1α*, and the sequences of the primers used are shown in Table S9 [79]. The detailed methods and steps can be found in our previous publication [39].

## 5. Conclusions

We identified miRNAs in the phloem and developing xylem of *P.* *deltoides* via small RNA sequencing and target genes of miRNAs via degradome sequencing. We found that most target genes of miRNAs were transcription factors. Furthermore, 13 and nine miRNAs were found to be involved in lignin and cellulose biosynthesis, respectively. This study provides new insights into the wood formation of poplar.

## Figures and Tables

**Table 1:** Summary of sRNA libraries.

Sample	Raw Reads	Clean Reads	Q30 (%)	GC (%)	Mapped Rate (%)
P1	11,419,418	11,161,174	95.96	50.74	90.07
P2	11,768,425	11,432,450	95.47	51.42	95.10
P3	11,997,478	11,760,792	95.83	50.77	91.37
X1	10,320,359	10,100,623	95.85	50.13	97.40
X2	10,796,007	10,499,531	95.17	50.58	97.22
X3	11,069,777	10,824,465	95.52	50.27	97.39

**Table 2:** Target genes of miRNAs identified in the phloem and developing xylem of *P.* *deltoides* by degradome sequencing.

miRNA	Transcript Annotation	Degradome Category	Degradome *p* Value
miR171a-mature	GRAS	0	0.000271151
miR160f-mature	ARF17	0	0.000271151
miR167d-mature	ARF8	0	0.000271151
miR396b-mature	GRF4	0	0.000271151
miR472a-star	AGO3	0	0.000271151
miR319a-mature	MYB65	0	0.000542229
miR164d-mature	NAC80	0	0.000542229
miR172c-mature	AP2	0	0.000813233
miR160d-mature	ARF10	0	0.002708204
miR160e-mature	ARF16	0	0.002708204
miR396a-mature	GRF9	0	0.002708204
miR530-mature	bHLH	0	0.017206273
miR396d-mature	SCL13	0	0.019336135
miR171d-star	WRKY	1	0.027125399

Note: In this study, we used Cleaveland4 software to predict target gene of miRNAs via degradome sequencing, which classifies the target genes confidence (Category) into five categories, with smaller categories generating more reliable results. Category0 represents the highest abundance of degradome sequencing reads matched to this target site and the site is unique, as can be seen from the Target plot where a sharp peak is formed at Cateogry0; Category1 represents the highest abundance of degradome sequencing reads matched to the target site but the site is not unique; Category2 represents an above average abundance of degradome sequencing reads matched to the target site; Category3 represents the below average abundance of degradome sequencing reads matched to the target site; and Category4 means that only one of the degradome sequencing reads matched the target site. Category3 or 4 means that the results are not very reliable and often need to be filtered out.

## Data Availability

All the data are shown in the main manuscript and in the Supplementary Materials. The sRNA sequencing described in this manuscript were submitted to the National Center for Biotechnology Information (NCBI) under accession codes PRJNA818493.

## Supplementary Materials

The following supporting information can be downloaded at: https://www.mdpi.com/article/10.3390/ijms23094537/s1.
