# Supplementary material for: Integrated Degradome and Srna Sequencing Revealed miRNA-mRNA Regulatory Networks between the Phloem and Developing Xylem of Poplar
Source: Int J Mol Sci. 2022 Apr 20;23(9):4537. doi: 10.3390/ijms23094537 (PMC9100975; doi:10.3390/ijms23094537)
Supplement: Supplementary file 1 [file ijms-23-04537-s001.zip › Figure S1-Figure S5.pdf]

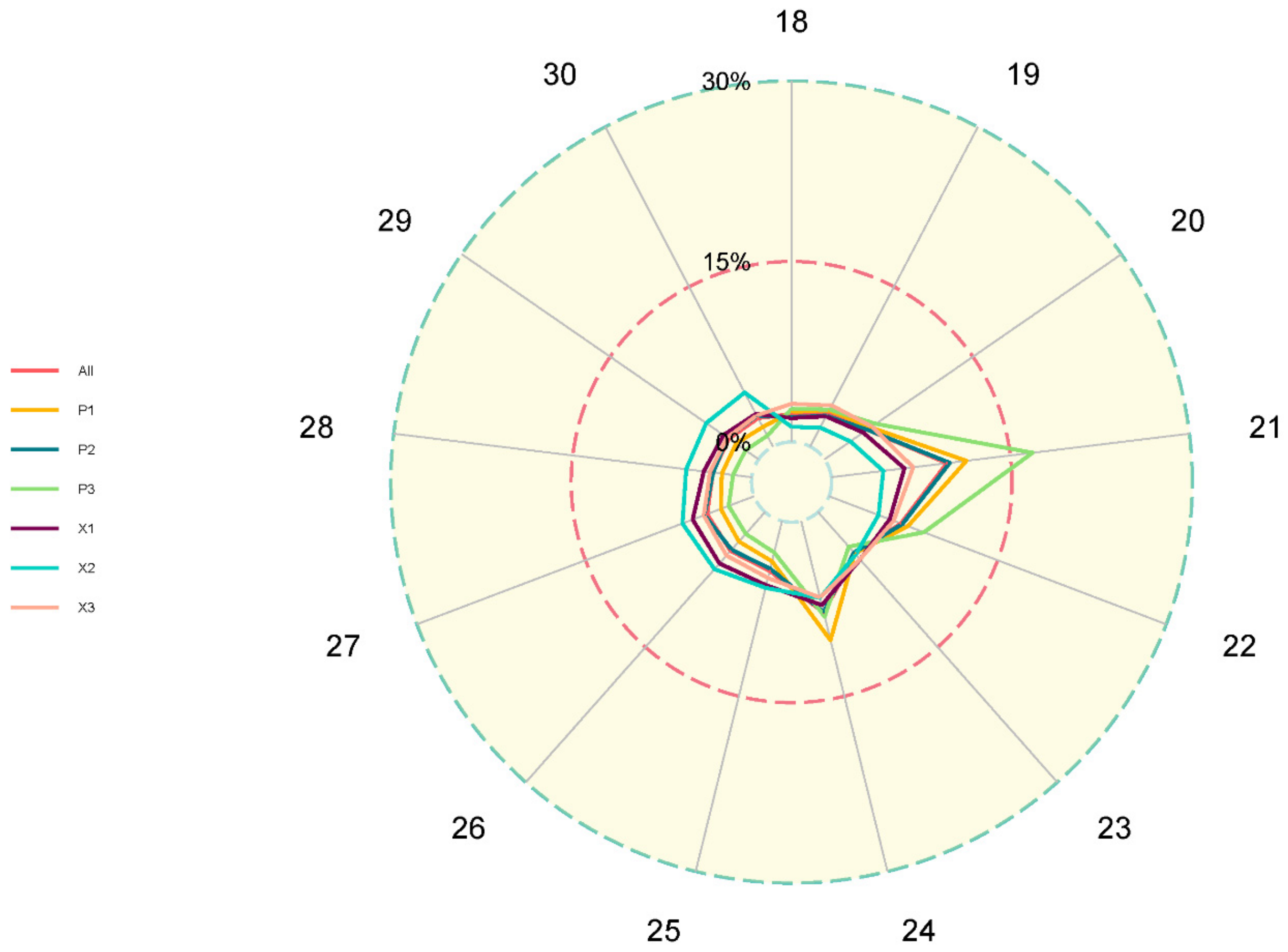

Figure S1 Length distribution of six sRNA libraries

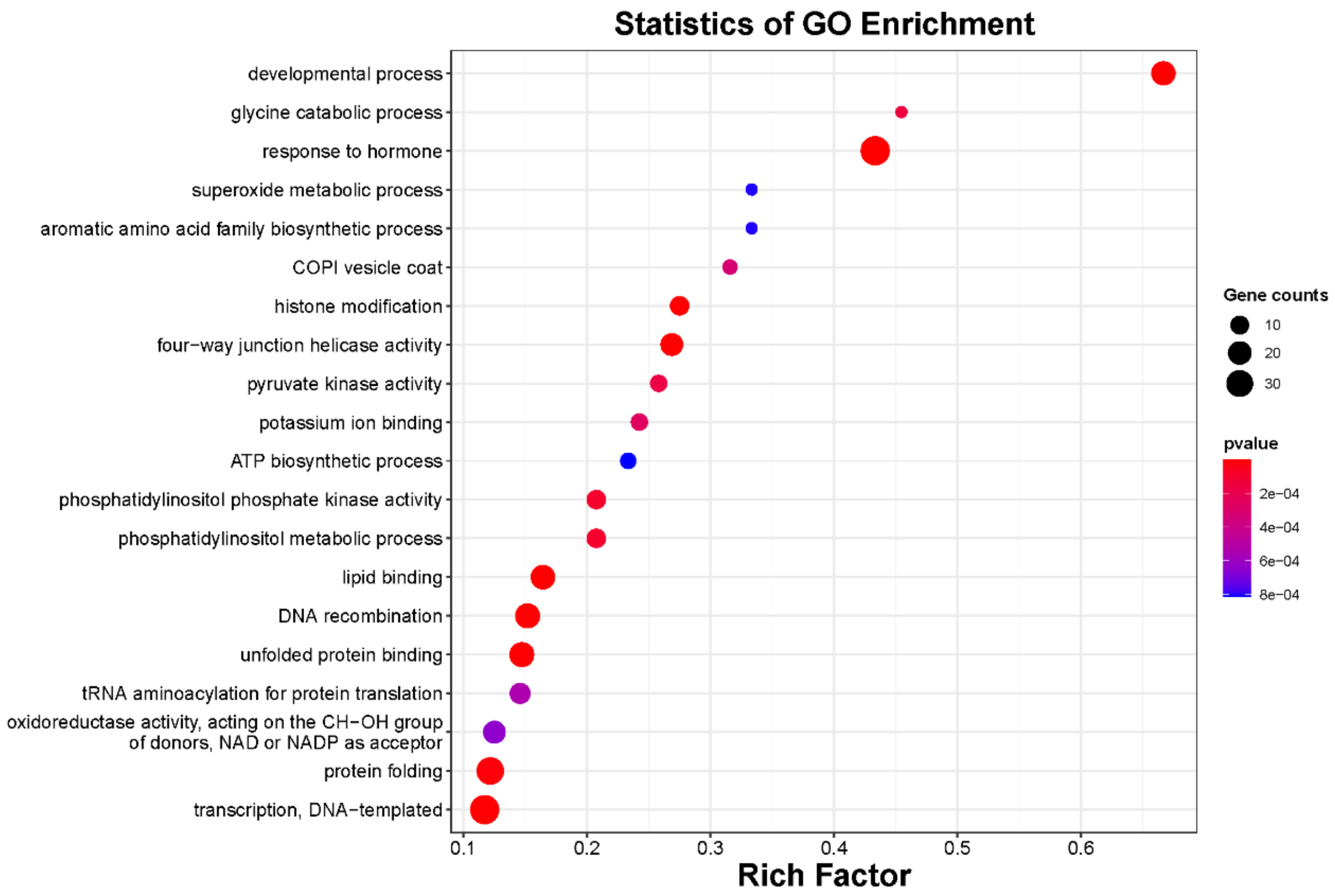

Figure S2 GO enrichment statistics of miRNA target genes

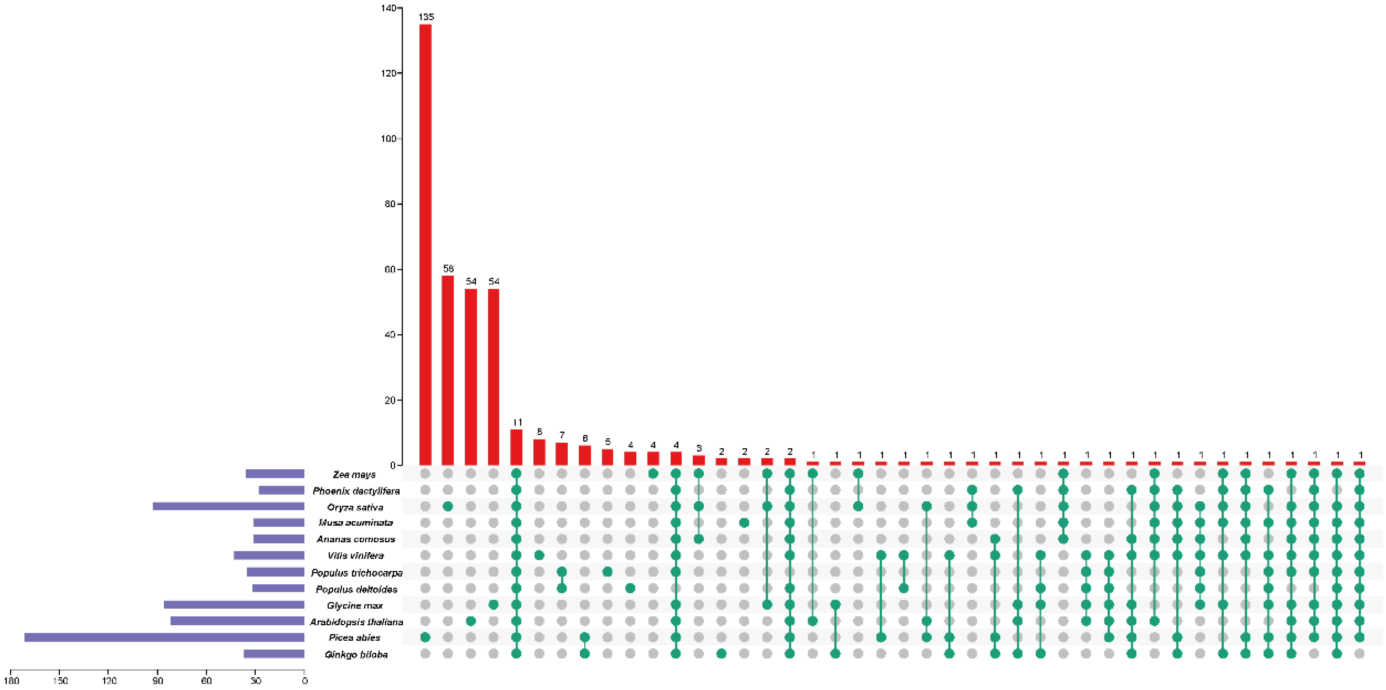

Figure S3 MiRNA families in 12 plants

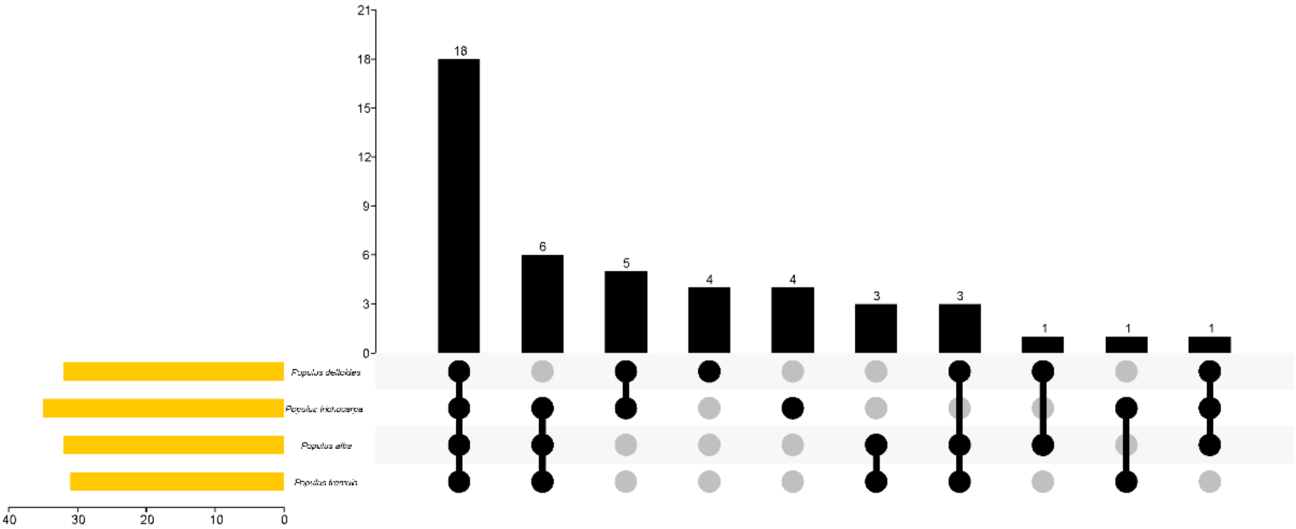

Figure S4 MiRNA families in four species of Populus

(*P. deltoides*, *P. trichocarpa*, *P. alba*, and *P. tremula*)

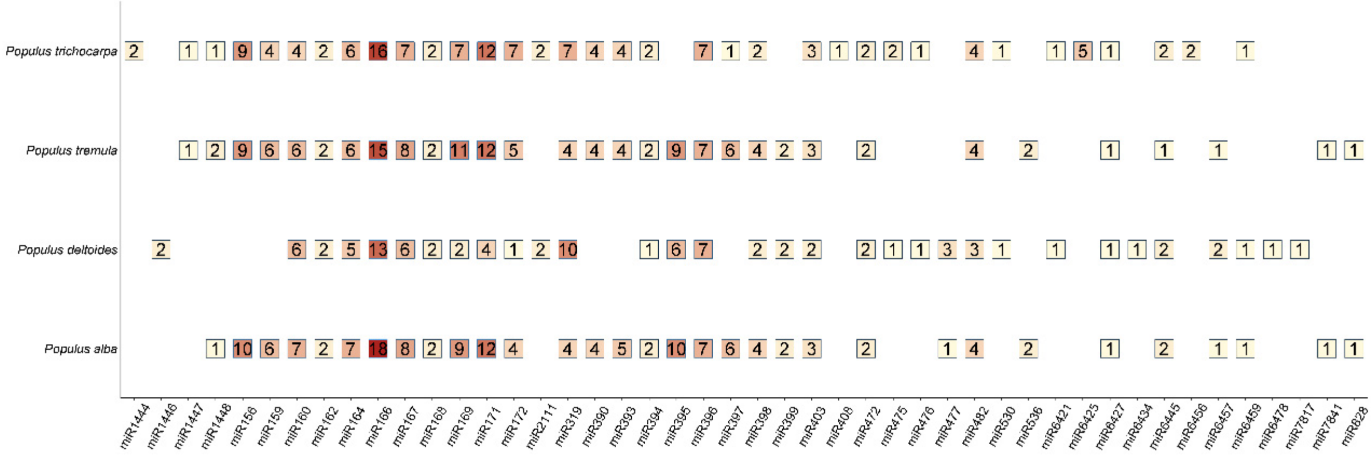

Figure S5 MiRNA family members in four species of Populus

(*P. deltoides*, *P. trichocarpa*, *P. alba*, and *P. tremula*)
